# ﻿A new species of anthothelid octocoral (Cnidaria, Alcyonacea) discovered on an algal reef of Taiwan

**DOI:** 10.3897/zookeys.1089.77273

**Published:** 2022-03-16

**Authors:** Tzu-Hsuan Tu, Chang-Feng Dai

**Affiliations:** 1 Department of Oceanography, National Sun Yat-sen University, Kaohsiung 804, Taiwan National Sun Yat-sen University Kaohsiung Taiwan; 2 Institute of Oceanography, National Taiwan University, Taipei 106, Taiwan National Taiwan University Taipei Taiwan

**Keywords:** *28S rDNA*, Anthothelidae, *cox2*-IGR-*cox1*, molecular phylogeny, *msh1*, northwestern Pacific, Scleraxonia

## Abstract

A molecular phylogenetic analysis of 132 octocoral species reveals a close relationship between specimens collected from the intertidal pools of the Datan Algal Reef, Taoyuan, Taiwan, and *Erythropodiumcaribaeorum* (Duchassaing & Michelotti, 1860), but the two species have distinct morphological features. On the basis of morphological differences in polyps and sclerites, we identify and describe a new *Erythropodium* species: *E.taoyuanensis***sp. nov.** The distinct identifying features of *E.taoyuanensis***sp. nov.** include the upright contractile polyps from thin encrusting membranes and abundant 6-radiate sclerites. Using an integrative approach, we present the findings of morphological comparisons and molecular phylogenetic analyses to demonstrate that *E.taoyuanensis***sp. nov.** is distinct from other *Erythropodium* species. Our study contributes to the knowledge of octocoral biodiversity in marginal habitats.

## ﻿Introduction

The Datan Algal Reef located in northwestern Taiwan, which occupies the intertidal flat toward the sublittoral along the 27-km long coastline of Taoyuan City, is composed of crustose coralline algae. Both sandy and muddy habitats occur in a mosaic pattern within the algal reef (Kuo et al. 2020). The porous algal reefs host a relatively high benthic diversity and biomass, such as crustaceans, polychaetes, and sipunculans (Lin 2020). Because of the geographic location and availability of hard substratum, the Datan Algal Reef may be a stepping stone to connecting reef-associated species between tropical corals and non-reefal coral communities in the Taiwan Strait (Chen 2017), while the physical environment in the algal reef may be considered a marginal habitat for most corals (Kuo et al. 2020). The sedimentation rates in the Datan Algal Reef are extremely high, ranging between 3,818 and 29,166 mg cm^−2^ day^−1^ (Kuo et al. 2020), which far exceeds the rate (10 mg cm^−2^ day^−1^) in a healthy shallow water tropical to subtropical coral reef (Rogers 1990). Therefore, the water column in rock pools is turbid and contains a high concentration of sand and particles formed by erosion, wave action, and tidal currents. Although the physical conditions may deter most corals, a stable population of the caryophyllid coral *Polycyathuschaishanensis* Lin et al., 2012 live in the tidal pool off the Datan Algal Reef (Kuo et al. 2020).

Meanwhile, the Datan Algal Reef is currently facing destruction from the development and construction of liquefied natural gas (**LNG**) storage terminals and ports by the Taiwan Chinese Petrol Corporation (**CPC**). Therefore, multiple environmental impact assessment surveys have been conducted. The intertidal surveys led to the discovery of a species of *Erythropodium* Kölliker, 1865 in the tidal pools (Lin 2020).

*Erythropodium* is a genus of shallow water soft corals forming endosymbiotic association with Symbiodiniaceae belonging to the family Anthothelidae Broch, 1916. Although it is widely distributed from tropical to temperate regions, its populations are not abundant (Bayer 1961). *Erythropodium* has been documented in a relatively small and fragmented geographical range, including the Caribbean Sea, the Southwestern Atlantic, northern Australia, and the Solomon Islands, with only three nominal species recorded worldwide (Duchassaing and Michelotti 1860; Bayer 1961; Utinomi 1971; Carpinelli et al. 2020). Furthermore, *Erythropodium* has not been recorded in the North Pacific Ocean. Its traditional diagnostic morphological features include thick encrusting sheet-like colonies without conspicuous upright lobes or branches, predominant 6-radiate sclerites, and a purplish red coenenchyme surface (Kölliker 1865; Bayer 1961, 1981) separate it from other genera within Anthothelidae. *Erythropodiumcaribaeorum* (Duchassaing & Michelotti, 1860), the type species of this genus is originally distributed in the Caribbean Sea and has invaded into the Southwestern Atlantic Ocean (Carpinelli et al. 2020). The other two species only reported in their type locality include *E.salomonense* Thomson & Mackinnon, 1910 in the Indian Ocean and *E.hicksoni* (Utinomi, 1971) in the south Pacific Ocean. Here, we describe and illustrate an additional species, *E.taoyuanensis* sp. nov. The freshly collected material was also subjected to molecular phylogenetic analyses, the results of which substantiated the taxonomic findings that led us to assign the new *Erythropodium* species.

## ﻿Materials and methods

### ﻿Collection and morphological analysis

Based on an environmental impact assessment report, the Datan Algal Reef, Taoyuan, Taiwan was divided into two subsections, Datan G1 and Datan G2 (Kuo et al. 2020). Collection and observation were conducted in Datan G2, during the spring low tide on June 24, 2021. Specimens were collected by reef walking and stored in seawater. After collection, one of the specimens (NMMB-CR000148) was preserved in absolute ethanol, and the remaining specimens were maintained in seawater with the addition of magnesium chloride overnight and then preserved in 75% ethanol. The holotype and paratypes are deposited at the National Museum of Marine Biology and Aquarium, Pingtung County, Taiwan (**NMMB-CR**). Selected fragments from four specimens were dissolved in sodium hypochlorite to examine sclerites under both light microscope and scanning electron microscope (S-3000N, Hitachi, Japan).

### ﻿Molecular phylogenetic analysis

Polyps from four colonies (NMMB-CR000148 to NMMB-CR000151) were used to extract DNA. DNeasy PowerSoil Kit (Qiagen, CA, USA) was used for DNA extraction, according to the manufacturer’s protocol. The primer pair COII8068XF and COIoctR was used to amplify *cox2*-IGR-*cox1* (France and Hoover 2002; McFadden et al. 2011). Furthermore, we designed a new primer pair (MSH-Antho-F: ARTTCTATGAACTTTGGCATGAGC and MSH-Antho-R: YTAGCATVGGGTTCAGAGGG) from sequences of Anthothelidae including *Erythropodium*, *Anthothela*, and *Iciligorgia* to amplify partial *mtMutS* region. The nuclear *28S rDNA* was amplified according to Halàsz et al. (2015), using the primers 28S-Far and 28S-Rab (McFadden and van Ofwegen 2013). The amplicons were purified and further sequenced using the ABI 3730 DNA Analyser. The sequences of NMMB-CR000148 were deposited in GenBank with accession numbers, OK480042, OK483343, and OK482879 for *cox2*-IGR-*cox1*, *mtMutS*, and *28S rDNA*, respectively and compared with sequences listed in McFadden and van Ofwegen (2012) and partial species in van der Ham et al. (2009) (Suppl. material 1: Table S1).

The obtained sequences were edited using Geneious Prime v. 2021.2.2 (Biomatters, New Zealand) aligned to data from McFadden and van Ofwegen (2012) and partial species in van der Ham et al. (2009) using MUSCLE alignment. Maximum-likelihood (ML) analyses were run using RAxML-NG v. 1.0.3 (Kozlov et al. 2019) with TVM+I+G and GTR+I+G models applied to mitochondrial genes and 28S rDNA, respectively. Bayesian inference (BI) was run using MrBayes v. 3.2.7 (Huelsenbeck and Ronquist 2001) with the same data partitions, while a GTR model was applied separately to each partition because MrBayes does not support the TVM model. Topologies were edited using FigTree v. 1.4.4 (accessible at http://tree.bio.ed.ac.uk/software/figtree/). Because the stoloniferan genus *Cornularia* Lamarck, 1816 is the sister taxon to all other octocorals, the sequences of *C.cornucopiae* (Pallas, 1766) and *C.pabloi* McFadden & van Ofwegen, 2012 were used as outgroups to root the phylogenetic trees (McFadden and van Ofwegen 2012).

## ﻿Results

### ﻿Taxonomy

The following key used to identify species of *Erythropodium* is based on the original descriptions of *E.caribaeorum*, *E.hicksoni*, and *E.salomonense* (Duchassaing and Michelotti 1860; Thomson and Mackinnon 1910; Utinomi 1971), Bayer’s (1961) description of *E.caribaeorum*, and the direct examation of type specimens of the new described *E.taoyuanensis* sp. nov.

### ﻿Key to species of *Erythropodium*

**Table:** 

1	Coenenchyme thin generally < 1 mm. Polyps contractile, do not fully retract into coenenchyme	***E.taoyuanensis* sp. nov.**
–	Coenenchyme thick generally > 1 mm. Polyps retractile, fully retract into coenenchyme	**2**
2	Sclerites in the form of rod present	***E.caribaeorum***
–	Sclerites in the form of rod absent	3
3	Coenenchymal sclerites are capstan-like triradiates or tetraradiates	***E.hicksoni***
–	Coenenchymal sclerites are double-spheres	***E.salomonense***

### ﻿Systematics

#### Class Anthozoa Ehrenberg, 1831

**Subclass Octocorallia Haeckel, 1866**

**Order Alcyonacea Lamouroux, 1812**

**Family Anthothelidae Broch, 1916**

##### Genus *Erythropodium* Kölliker, 1865

###### ﻿ Erythropodium taoyuanensis

Taxon classificationAnimaliaAlcyonaceaAnthothelidae

0CD54755-1DAB-5D52-85C8-EEA3FB555287

http://zoobank.org/A83374ED-B308-4C8C-9708-531A5A32840C

Figs 1 , 2 , 3 , 4

####### Material examined.

***Holotype*.** Taiwan, Taoyuan, Datan Algal Reef; 25°02'7.849"N, 121°02'56.059"E; −30 cm (below sea level); 24 Jun. 2021; T.-H. Tu and E.-J. Lin leg.; tidal pool, hand collecting; GenBank: OK480042, OK483343, and OK482879; NMMB-CR000148.

***Paratype*.** Taiwan; same data as holotype; NMMB-CR000149.

####### Other material.

Taiwan; same data as holotype; 21 Sep. 2020; NMMB-CR000150.Taiwan; same data as holotype; 21 Sep. 2020; M.-H. Lin and L.-C. Liu leg.; NMMB-CR000151.

####### Diagnosis.

The holotype colony is composed of upright polyps arising separately from a encrusting membrane less than 1 mm thick or a network of ribbon-like stolons. When fully extended, polyps are around 3 mm long, and the tentacles are slender with 10–13 pairs of pinnules on either side of the rachis. Polyps are contractile and cannot fully retract into the basement layer. Sclerites are mostly 6-radiate sclerites, with a few being irregular radiates. When alive, polyps are yellowish pink, and the basement layer is magenta.

####### Description of the holotype.

**Figure 1.**
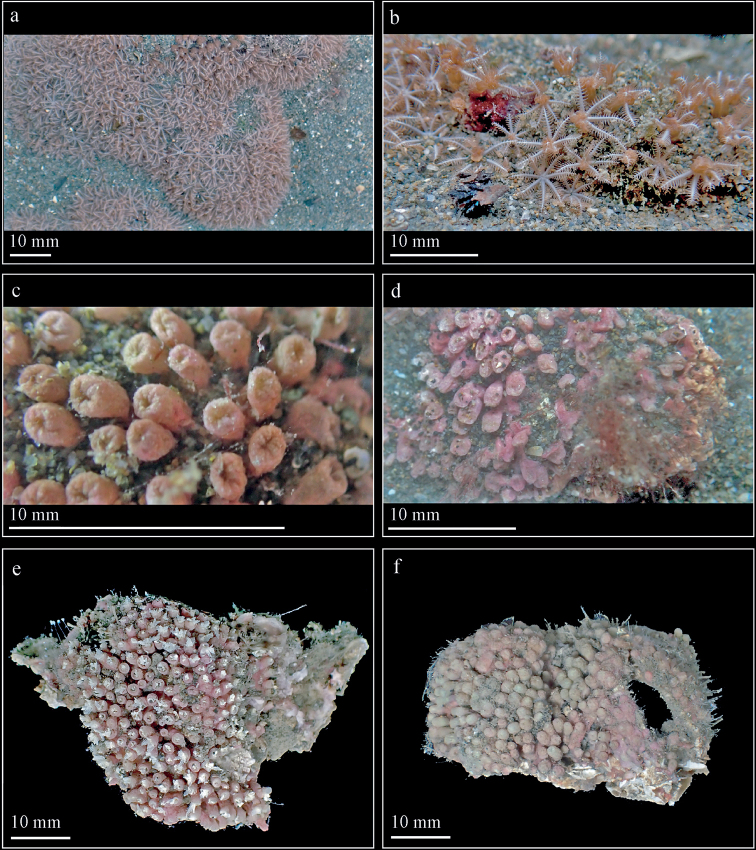
*Erythropodiumtaoyuanensis* sp. nov. **a** intertidal population *in situ***b** close-up of **a c, d** contracted polyps *in situ***e** holotype (NMMB-CR000148) in preserved state **f** paratype (NMMB-CR000149) in preserved state.

**Figure 2.**
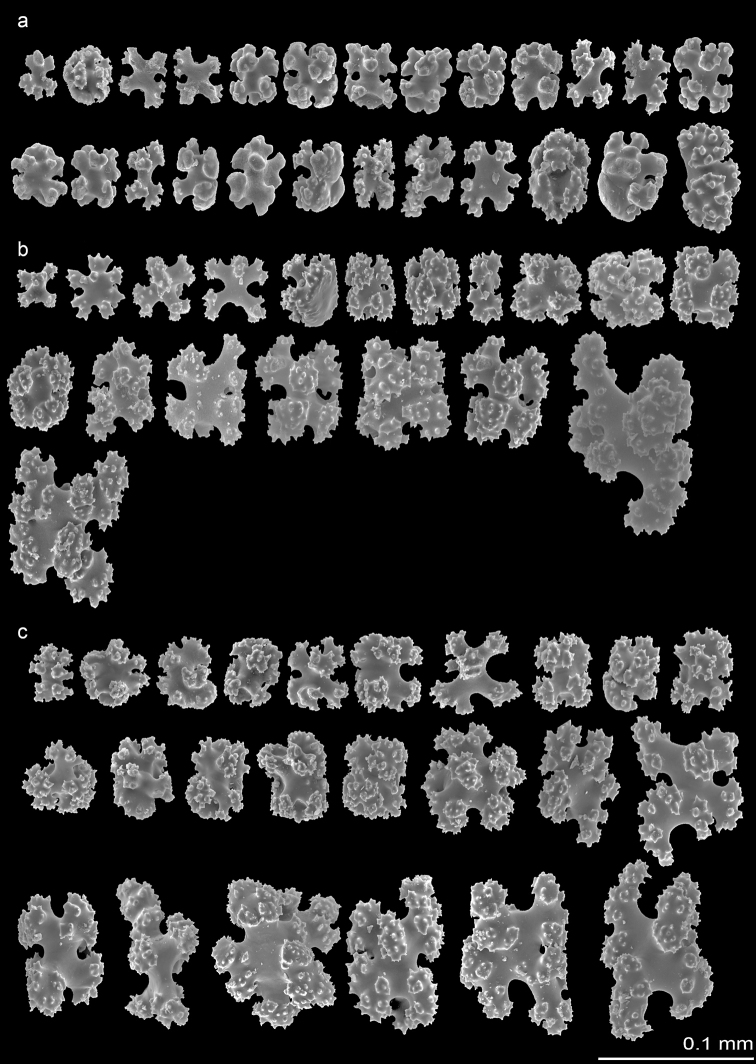
*Erythropodiumtaoyuanensis*, holotype, NMMB-CR000148 **a** sclerites of the polyp **b** sclerites of the polyp wall **c** sclerites of the cortex.

(Figs 1, 2). ***Colonial morphology*.** The holotype is an encrusting colony and attaching on barnacles and coralline algal substrate. When alive, the colony consisted of densely distributed polyps, up to 20/cm^2^, arising from the basement layer, which is completely covered by sand (Fig. 1a). In its preserved state, the holotype measures 57.0 mm × 33.8 mm × 22.8 mm. The thickness of the basal membrane in the alcohol-preserved holotype is less than 1 mm.

***Polyps*.** When fully extended, the polyps may attain approximately 2.5–3.0 mm in length (Fig. 1b). The fully spread tentacles are cylindrical, slender, and up to 2.5 mm × 0.8 mm, with 10–13 pairs of pinnules arranged in a single row on either side of the tentacle rachis (Fig. 1b). The polyps are contractile in both live and preserved state (Fig. 1); when contracted, they are cylindrical and measuring from the attachment at stolons to the tentacle base are around 1.5 mm in width (Fig. 1c, d). The pinnules (0.2–0.9 mm long) gradually taper at the end to a sharp tip. The polyps are associated with symbiotic unicellular algae.

***Sclerites*.**Sclerites are present in all parts of the holotype and evenly distributed in the coenenchyme, polyp body wall, tentacles, and pinnules. Six-radiate sclerites are the commonest type, representing more than 90% of sclerites in anthocodiae and tentacles. They are 0.032–0.068 mm in length and 0.025–0.036 mm in width with simple tubercles (Fig. 2a). The polyp wall contains abundant 6-radiate sclerites, derivatives of radiates which are 0.028–0.132 mm in length and 0.025–0.083 mm in width, with prominent tubercles and table-radiates (Fig. 2b). The average size of sclerites in the polyp wall is greater than that in the polyps. Sclerites in the cortex are similar to those of the polyp wall but larger in size, including 6-radiate sclerites (0.042–0.120 mm in length and 0.034–0.076 mm in width) and irregular radiates (0.046–0.080 mm in length and 0.100–0.130 mm in width) (Fig. 2c). Furthermore, some sclerites in the cortex are fused to form clumps.

***Color*.** In life, colors of tissue, autozooids, and cortical layer are translucent, white to yellowish, and pink to magenta, respectively. Under light microscope, sclerites are translucent, magenta, or reddish.

**Figure 3.**
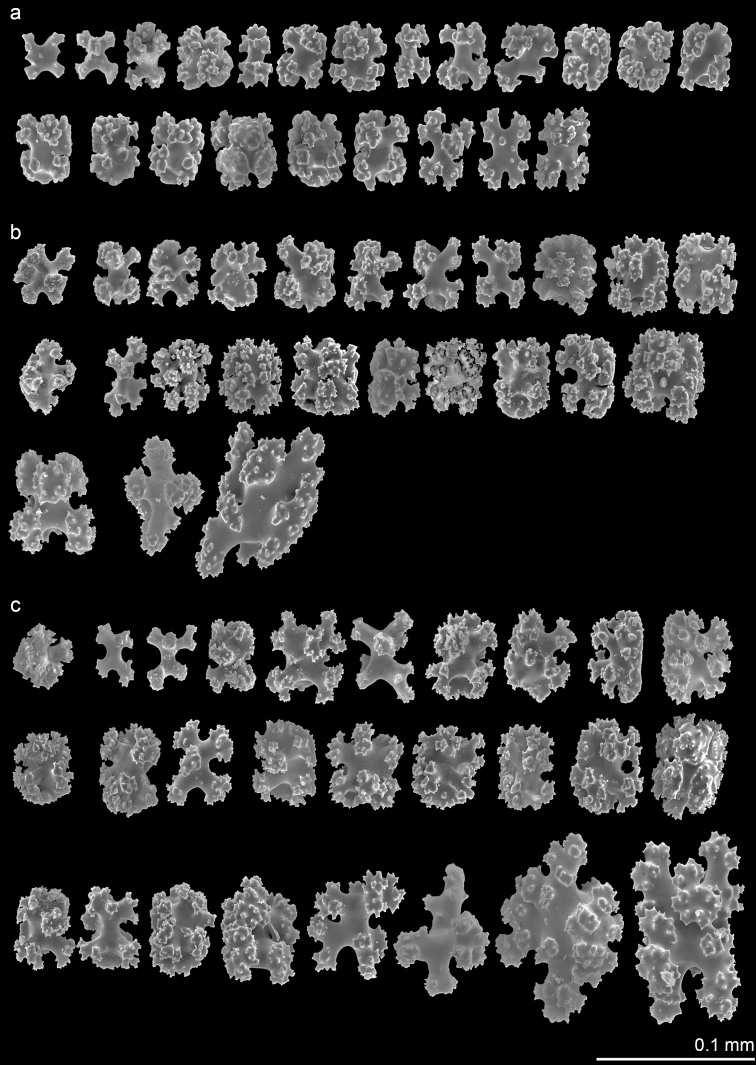
*Erythropodiumtaoyuanensis*, paratype, OCT133 NMMB-CR000149 **a** sclerites of the polyp **b** sclerites of the polyp wall **c** sclerites of the cortex.

**Figure 4.**
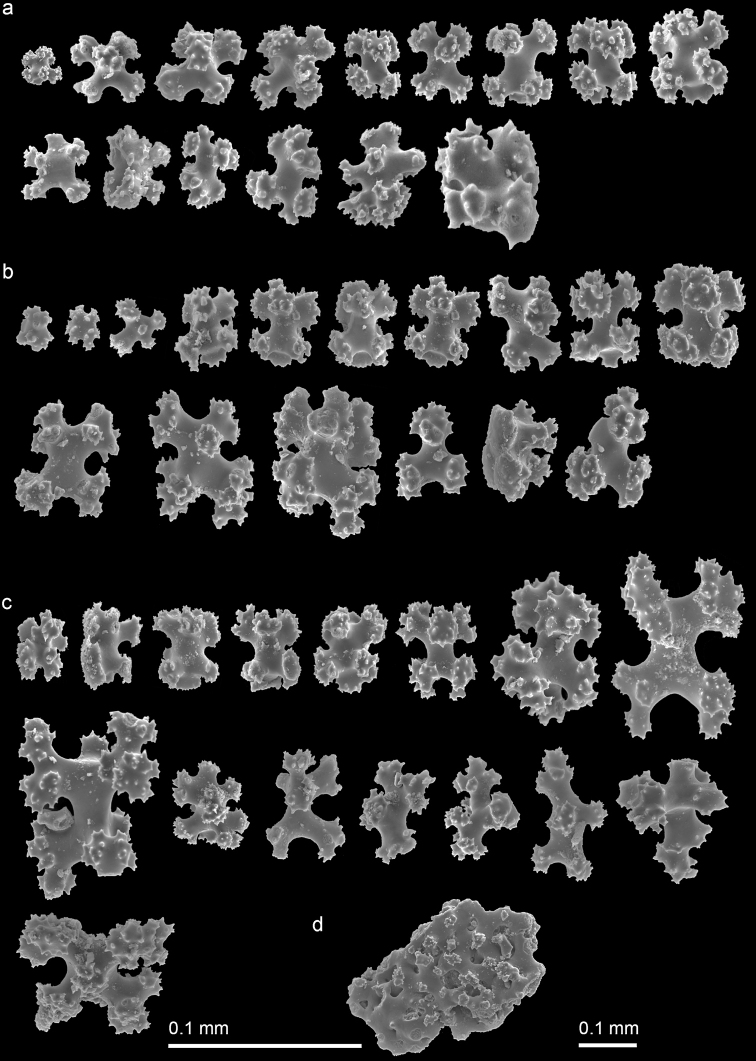
*Erythropodiumtaoyuanensis*, NMMB-CR000151 **a** sclerites of the polyp **b** sclerites of the polyp wall **c** sclerites of the cortex **d** fused sclerites in the cortex.

***Variation*.** Paratype (NMMB-CR000149) and non-type specimens (NMNB-CR000150 and NMNB-CR000151) show variation in the density of polyps ranging 5–20/cm^2^. Six-radiate sclerites are the commonest type of sclerites in the examined specimens, while their sizes are varied not only in different parts of a colony but also differ from what was observed in the holotype and across the specimens. The length and width of 6-radiate sclerites in the examined specimens is 0.020–0.068 mm and 0.020–0.053 mm, respectively, in polyp tissue; 0.024–0.098 mm and 0.018–0.070 mm, respectively, in polyp wall; and 0.022–0.118 mm and 0.026–0.075 mm, respectively, in cortex (Figs 3, 4). All examined specimens possess similar diagnostic features as the holotype from the level of colony to sclerits including upright polyps arising from a encrusting membrane, contraticle polyps, and predominant six-radiate sclerites. The major differences between examined specimens are reflected in the density and size variation of polyps and sclerites, respectively.

####### Differential diagnosis.

When comparing the morphology of *E.taoyuanensis* sp. nov. to the other three *Erythropodium* species, basal membrane, pinnule arrangement, retractile or contractile ability of polyps, and shape and size of sclerites (Table 1) were examined, with the contractibility of polyp and shape of sclerites considered as the most distinct characters.

**Table 1.** Diagnostic traits of nominal *Erytropodium* species.

Species name	Diagnostic traits				
	Colony	Coenenchyme	Polyp	Sclerite	References
*Erythropodiumcaribaeorum*	Encrusting, membranous carpet-like colony	Thick cortical layer, ~3 mm	Retractile polyps with elongated pinnules arragned in a single pair of rows	Dominant 6-radiate sclerites and irregular radiate scleirtes	Duchassaing and Michelotti 1860: pl. I. figs 8–11; Bayer 1961: 75; fig. 16e–h; Carpinelli et al. 2020: 177; figs 1, 2
*Erythropodiumhicksoni*	Membranous colony	Thick cortical layer, ~3 mm	Retractile polyps with 9 pairs of pinnules per tentacle	Triradiates, quadriradiates, flattened rods, and spindles	Utinomi 1971: 8–10, fig. 2; pl. 7. fig. 3
*Erythropodiumsalomonense*	Encrusting form	Thick cortical layer, 1.5–2 mm	Retractile polyps	Spindles, double spheres, irregular sclerites	Thomson and Mackinnon 1910: 174–175; pl. 12, fig. 8; pl. 13, fig. 9
*Erythropodiumtaoyuanensis*	Encrusting, membranous carpet-like colony	Think cortical layer, generally < 1 mm	Contractile polyps with 10–13 pairs of pinnules per tentacle	Dominant 6-radiate sclerites and derivatives of radiates	Present study

According to Duchassaing and Michelotti (1860), Bayer (1961), and Carpinelli et al. (2020), the diagnostic features of *E.caribaeorum* include an encrusting and membranous carpet-like colony, retractile polyps, elongated pinnules, thick cortical layer, and predominantly 6-radiate sclerites (Table 1). While the colonies of *E.taoyuanensis* sp. nov. form firm expansions on rocks similar to the colonial form of *E.caribaeorum*, the thinner cortical layer, shorter pinnules, and contractile polyps are distinct features. Furthermore, the types and shapes of radiates are the main features to distinguish these two species. Compared with *E.caribaeorum*, *E.taoyuanensis* sp. nov. possesses irregular radiates with generally enlarged tubercles having tiny protuberances. Compared with the creamy white and 3-mm-thick basal membrane in *E.hicksoni* (Table 1), the basal membrane in *E.taoyuanensis* sp. nov. is pink in the preserved state, similar to the color as in life, and thinner—generally less than 1 mm thick. Although pinnules in both species are arranged in a single pair of rows (one at each side of a tentacle), *E.hicksoni* normally has nine pairs of pinnules per tentacle, whereas *E.taoyuanensis* has 10–13 pairs. In addition, the polyps are retractile in *E.hicksoni* but contractile in *E.taoyuanensis*. Sclerites in *E.hicksoni* include triradiates, tetraradiates, flattened rods, and spindles. However, *E.taoyuanensis* sp. nov. has only 6-radiate sclerites. Finally, *E.salomonense* and *E.taoyuanensis* sp. nov. can be distinguished by the type and shape of the radiates. Additionally, the retractile polyps in *E.salomonense* (Table 1) are distinct from the contractile polyps in *E.taoyuanensis*. The above variations support that *E.taoyuanensis* is distinct from the other nominal *Erythropodium* species.

####### Etymology.

The specific name *taoyuanensis* alludes to the city’s name, Taoyuan, where the specimens were collected.

####### Distribution.

The Datan G2 in Datan Algal Reef, Taoyuan, Taiwan, is the only location where this species is known; it has a biodiverse coralline algal reef. *Erythropodiumtaoyuanensis* sp. nov. is one of the dominant sessile organisms encrusting the rocks at this location and is generally restricted to near the low tidal line, and it may be exposed to the air during the spring low tide.

### ﻿Phylogenetic analyses

**Figure 5.**
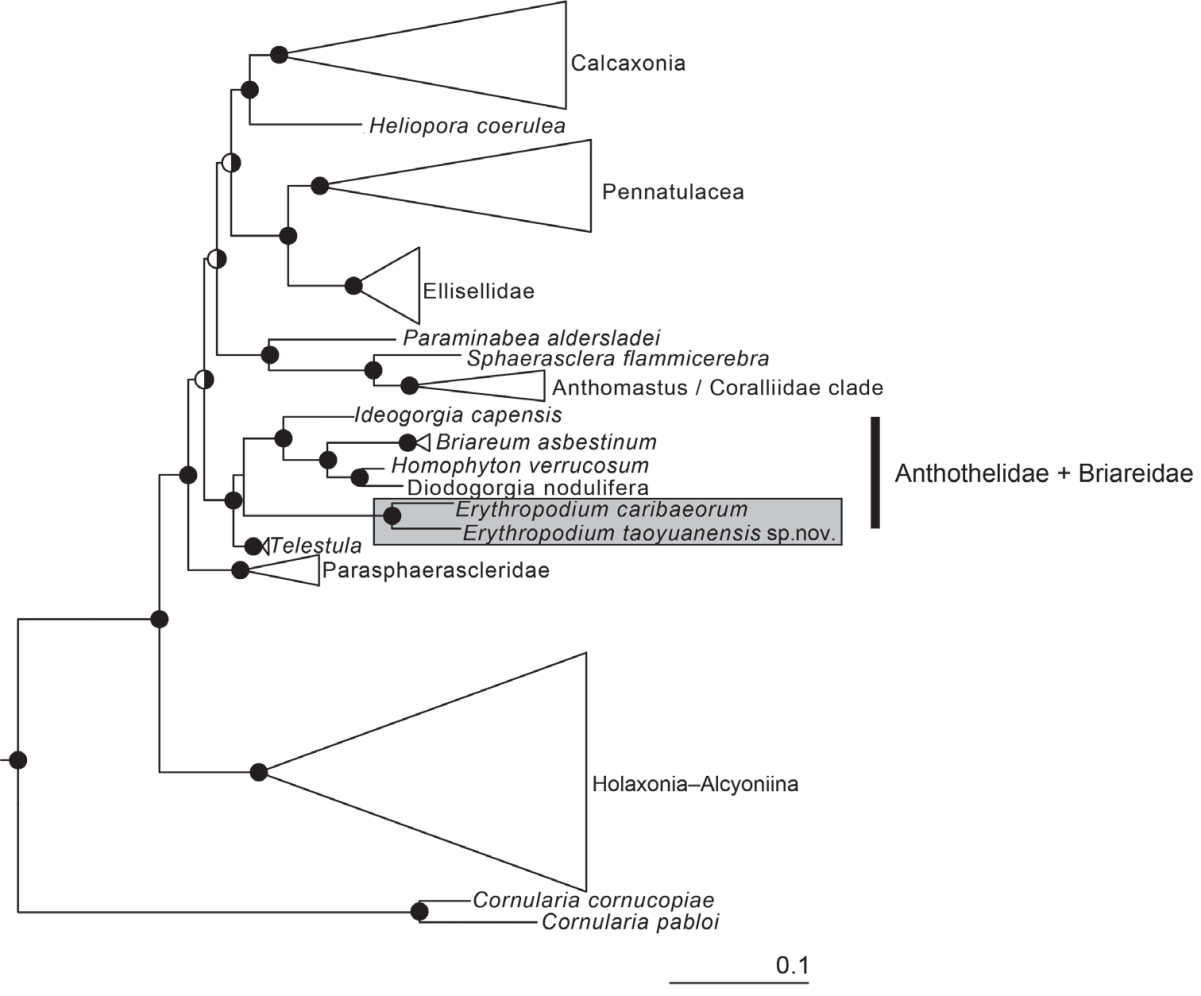
Phylogenetic relationship reconstruction (2543 nt of concatenated *msh1*, *cox2*-IGR-*cox1*, *28S rDNA*) of the Holaxonia–Alcyoniina clade of Octocorallia. Solid circles at nodes indicate strong support from both maximum-likelihood (bootstrap value > 70%) and Bayesian inference (posterior probability > 0.95); split circles indicate strong support from one analysis only (left half solid: supported by maximum-likelihood; right half solid: supported by Bayesian analyses).

Sequencing nuclear *28S rDNA*, and mitochondrial *cox2*-IGR-*cox1* and *msh1* resulted in 784, 777, and 585 bps, respectively, yielding a concatenated alignment of 2542 bps containing 1641 phylogenetically informative sites. All four *E.taoyuanensis* sp. nov. specimens in this collection had identical genotypes at the sequenced regions. The genetic distances (uncorrected *p*) between the specimens from the Datan Algal Reef and *E.caribaeorum* are 6.2% at *msh1*, 3.7% *cox2*-IGR-*cox1*, and 4.5% at 28S. As has been demonstrated previously based on analyses of similar datasets (McFadden and van Ofwegen 2012, 2013), both ML and BI indicated that the concatenated alignment supported the division of octocorals into two major clades: one composed of Holaxonia–Alcyoniina and the other composed of the majority of Calcaxonia, Pennatulacea, *Heliopora*, and Scleraxonia (Fig. 5). In the latter clade, the family Parasphaerascleridae McFadden & van Ofwegen, 2013 of Alcyonacea is strongly supported as the sister taxon to a group consisting of previously recognized Calcaxonia–Pennatulacea and *Anthomastus*–Coralliidae clades, and a small subgroup of a heterogenous mix of scleraxonians plus the stoloniferan genus *Telestula* Madsen, 1944 (Fig. 5). Both phylogenetic analyses placed specimens of *E.taoyuanensis* sp. nov. in the subgroup composed of heterogeneous scleraxonians including the genera *Erythropodium*, *Ideogorgia*, *Homophyton*, and *Diodogorgia* of Anthothelidae and *Briareum* of Briareidae (Fig. 5) with strong support (ML bootstrap = 100%; BI poster probability = 1.0). Within the subgroup, both ML and BI indicated that *E.taoyuanensis* sp. nov. is a sister taxon to *E.caribaeorum* (GenBank accession number: GQ342480, specimen RMNH.Coel. 40829).

## ﻿Discussion

*Erythropodiumtaoyuanensis* sp. nov. has only been discovered in the tidal pools at Datan G2 of the Datan Algal Reef. The tidal pool is periodically exposed to air and experiences variation in salinity, dissolved oxygen content, and temperature. Therefore, it is not a typical habitat for octocorals, and only a couple of species of *Sinularia* or *Asterospicularia* of Xeniidae have been observed in Taiwanese reefs (Dai 1991; Benayahu et al. 2004). By contrast, the low-water level also brings plentiful sunlight, which helps intertidal plant life grow quickly. In the Datan Algal Reef, the water column has a high sediment rate (Kuo et al. 2020). Therefore, living in tidal pools might help the zooxanthellate *E.taoyuanensis* overcome the turbid water. *Erythropodiumtaoyuanensis* sp. nov. is the first *Erythropodium* species identified to be distributed in the subtropical Indo-Pacific Ocean; other *Erythropodium* species have been reported in the Caribbean Sea, southwestern Atlantic, and temperate waters of the Indo-Pacific (Bayer 1961; Utinomi 1971; Carpinelli et al. 2020). This study is also the first to document an *Erythropodium* species off Taiwan. Meanwhile, the restricted distribution of *E.taoyuanensis* sp. nov. and members of *Erythropodium* in Taiwan further emphasize that their conservation is urgent. Unfortunately, their only known habitat, the Datan Algal Reef, is currently polluted by concrete from the construction of LNG receiving terminals and ports.

Morphologically, the specimens (NMMB-CR000148 to NMM-CR000151) collected from the Datan Algal Reef possessed the diagnostic feature of *Erythropodium*, such as thin, firm colony expansions on rocks and sclerites that are all derivatives of 6-radiate sclerites (Kölliker 1865; Bayer 1961). Therefore, they are considered to be an *Erythropodium* species, while the morphological features of polyps and composition of sclerites subsequently separate the specimens from the nominate species of *Erythropodium*. As the orginal descriptions of the three norminal *Erythropodium* species were based on the light-microsope observations and lacking definite figures representing diaonstic features, a future thorough redescription of the type specimens will contribute towards further identification of this genus. Molecular evidence has revealed that the genetic distances between the specimens from the Datan Algal Reef and *E.caribaeorum* are greater than general intraspecific variation of most octocorals, thereby further supporting that the specimens are a new *Erythropodium* species. Although *E.salomonense* and *E.hicksoni* were not included in our molecular analyses, the distinct morphological features still support the separation of *E.taoyuanensis* sp. nov. from the two nominal species. In summary, the new species described here is supported by both morphological and molecular evidence.

## Supplementary Material

XML Treatment for Erythropodium taoyuanensis

## Supplementary materials

Supplementary material 1 Table S1 Data type xlsx file Explanation note Sequnece used for phyloenetic reconstruction and their respective GenBank Accession numbers, according to McFadden & Ofwegen (2012). https://binary.pensoft.net/file/659361This dataset is made available under the Open Database License (http://opendatacommons.org/licenses/odbl/1.0/). The Open Database License (ODbL) is a license agreement intended to allow users to freely share, modify, and use this Dataset while maintaining this same freedom for others, provided that the original source and author(s) are credited.Tzu-Hsuan Tu, Chang-Feng Dai
